# Unified model involving genomics, magnetic resonance imaging and prostate‐specific antigen density outperforms individual co‐variables at predicting biopsy upgrading in patients on active surveillance for low risk prostate cancer

**DOI:** 10.1002/cnr2.1492

**Published:** 2021-12-20

**Authors:** Alp Tuna Beksac, Parita Ratnani, Zachary Dovey, Sneha Parekh, Ugo Falagario, Reza Roshandel, Stanislaw Sobotka, Deepshikha Kewlani, Avery Davis, Rachel Weil, Hafis Bashorun, Ivan Jambor, Sara Lewis, Kenneth Haines, Ashutosh K. Tewari

**Affiliations:** ^1^ Department of Urology Icahn School of Medicine at Mount Sinai New York USA; ^2^ Department of Radiology Icahn School of Medicine at Mount Sinai New York USA; ^3^ Department of Pathology Icahn School of Medicine at Mount Sinai New York USA

**Keywords:** active surveillance, biomarker, genomic prostate score, multiparametric MRI, oncotype, prostate cancer, PSA density

## Abstract

**Background:**

Active surveillance (AS) is the reference standard treatment for the management of low risk prostate cancer (PCa). Accurate assessment of tumor aggressiveness guides recruitment to AS programs to avoid conservative treatment of intermediate and higher risk patients. Nevertheless, underestimating the disease risk may occur in some patients recruited, with biopsy upgrading and the concomitant potential for delayed treatment.

**Aim:**

To evaluate the accuracy of mpMRI and GPS for the prediction of biopsy upgrading during active surveillance (AS) management of prostate cancer (PCa).

**Method:**

A retrospective analysis was performed on 144 patients recruited to AS from October 2013 to December 2020. Median follow was 4.8 (IQR 3.6, 6.3) years. Upgrading was defined as upgrading to biopsy grade group ≥2 on follow up biopsies. Cox proportional hazard regression was used to investigate the effect of PSA density (PSAD), baseline Prostate Imaging‐Reporting and Data System (PI‐RADS) v2.1 score and GPS on upgrading. Time‐to‐event outcome, defined as upgrading, was estimated using the Kaplan–Meier method with log‐rank test.

**Results:**

Overall rate of upgrading was 31.9% (n = 46). PSAD was higher in the patients who were upgraded (0.12 vs. 0.08 ng/ml^2^, *p* = .005), while no significant difference was present for median GPS in the overall cohort (overall median GPS 21; 22 upgrading vs. 20 no upgrading, *p* = .2044). On univariable cox proportional hazard regression analysis, the factors associated with increased risk of biopsy upgrading were PSA (HR = 1.30, CI 1.16–1.47, *p* = <.0001), PSAD (HR = 1.08, CI 1.05–1.12, *p* = <.0001) and higher PI‐RADS score (HR = 3.51, CI 1.56–7.91, *p* = .0024). On multivariable cox proportional hazard regression analysis, only PSAD (HR = 1.10, CI 1.06–1.14, *p* = <.001) and high PI‐RADS score (HR = 4.11, CI 1.79–9.44, *p* = .0009) were associated with upgrading. A cox regression model combining these three clinical features (PSAD ≥0.15 ng/ml^2^ at baseline, PI‐RADS Score and GPS) yielded a concordance index of 0.71 for the prediction of upgrading.

**Conclusion:**

In this study PSAD has higher accuracy over baseline PI‐RADS score and GPS score for the prediction of PCa upgrading during AS. However, combined use of PSAD, GPS and PI‐RADS Score yielded the highest predictive ability with a concordance index of 0.71.

## 1 INTRODUCTION

Active surveillance (AS) is the reference standard treatment for the management of low risk prostate cancer (PCa). Generally, AS programs require serial digital rectal examination (DRE) and prostate specific antigen (PSA) measurement, annual multi‐parametric Magnetic Resonance Imaging (mpMRI) of the prostate and repeat biopsies 2–3 years after diagnosis.^1^ This approach defers definitive treatment, providing significant quality of life benefits for those low risk patients recruited. Furthermore, disease progression and metastatic rate are extremely low.^2^ Despite promising evidence of safe long term inclusion for AS patients, up to 43.6% still require definitive treatment in 5 years, based on a review of over 10 000 men in AS programs across 12 countries.^3^

A more accurate assessment of tumor aggressiveness can also guide recruitment to AS programs to avoid conservative treatment of intermediate and higher risk patients. There are commercially available genomic tests that predict treatment outcome in prostate cancer.^4^ A 17 gene RNA expression assay known as the Oncotype Genomic prostate score (GPS) (Exact Science Corp.) is one such test that has been validated in a biopsy setting to predict treatment outcomes following radical prostatectomy (RP),^5^ as well as contributing to the decision for AS recruitment in newly diagnosed PCa patients.^6^, ^7^

One major concern for AS management lies in underdiagnosis of clinically significant prostate cancer (csPCa), with subsequent underestimation of disease risk.^8^ The term “clinically significant prostate cancer” (csPCa) has gained increasing acceptance with the widespread adoption of pre‐biopsy mpMRI, and refers to the detection of intermediate and high risk prostate cancer that would necessarily require radical treatment, rather than the detection of low risk prostate cancer which has AS as the standard of care. The ability of mpMRI to detect suspicious lesions with a greater chance of harboring csPCa, has improved clinical staging as well as the accuracy of repeat prostate biopsies via targeted biopsy (TB).^9^ mpMRI and TB show promise in the AS setting, however its routine use in AS has not yet been established.^10^ In this study, we sought to evaluate the individual roles of GPS and mpMRI in predicting upgrading during AS, as well as exploring the use of a unified model including both these co‐variables and PSAD.

## 2 PATIENTS AND METHODS

### 2.1 Study population

**FIGURE 1:**
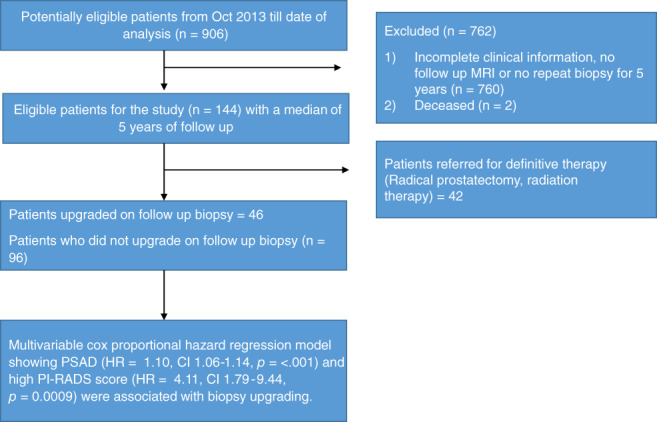
Flow Chart to define participant inclusion and exclusion criteria

An institutional review board approved retrospective analysis was performed on 144 patients who were diagnosed with National Comprehensive Cancer Network (NCCN) low risk, and low volume intermediate risk PCa and followed by the AS protocol from October 2013 to December 2020 (see Figure 1, flow chart). All patients had available GPS data, baseline mpMRI data and at least two follow up biopsies which may have included a confirmatory biopsy. Median follow up was approximately 4.8 (IQR 3.6, 6.3) years. Patients were diagnosed with systematic biopsy with or without TB, and second biopsy was defined as subsequent biopsy 12 months after the initial diagnostic biopsy. Repeat biopsies prior to 12 months from diagnostic biopsy were regarded as “confirmatory” (17/144 patients [11.8%]).

### 2.2 Imaging and biopsy protocol

All patients with low risk PCa underwent a baseline mpMRI performed as a part of routine clinical practice from September 2011 to November 2020 at different institutions using different mpMRI acquisition protocols. A commonly used imaging acquisition protocol consisted of axial, sagittal, coronal T2‐weighted imaging, diffusion‐weighted imaging and dynamic contrast enhancement performed using intravenous contrast. mpMRI studies for the first 75 patients (52%; baseline MRI performed between September 2011 and September 2018) were anonymized and reported by one reader (I.J., 9 years of prostate MRI experience) in random order using the PI‐RADSv2.1^11^ without knowledge of clinical, laboratory or biopsy findings. The presence of MRI Extracapsular Extension (ECE) or Seminal Vesicle (SVI) on baseline MRI were exclusion criteria for recruitment to AS. Clinical reports for the remaining 69 patients (47.9%; baseline MRI performed between October 2018 and November 2020) were read by different radiologists with genitourinary subspecialist interest. All patients with MRI lesions defined as PI‐RADS ≥3 were offered TB. TB was performed by baseline memory fusion with real time transrectal ultrasound images (Artemis, Eigen, Grass Valley, CA, USA). Three to five samples were taken from each target lesion and standard 12‐core template sampling was performed in addition to the target lesion(s) biopsies.

The AS protocol included PSA and DRE every 3–6 months and annual mpMRI. Patients received follow up biopsy if they had a new positive DRE finding, rapid increase in PSA as well as a new or a change in dimensions of PI‐RADS >3 lesions. In the absence of these clinical risk factors, every patient underwent 12‐core standard biopsy every 3 years.

### 2.3 Variables and outcome

Baseline clinical and pathological data included age, race, BMI, PSAD, biopsy Gleason score, PI‐RADS Score, GPS and GPS risk group. For PSAD measurement, the volume data were defined by baseline mpMRI measurement. Patients were stratified by PI‐RADS score (1, 2, and 3 grouped as low risk vs. 4 and 5 grouped as high risk) and GPS defined as low risk <18, intermediate risk 18–30 or high risk 31–100. Upgrading was defined as upgrading to grade group ≥2 or higher on follow up biopsies. Upgrading was the primary outcome of the study and resulted in patients being offered definitive therapy, via RP or external beam radiation.

### 2.4 Statistical analysis

**FIGURE 2:**
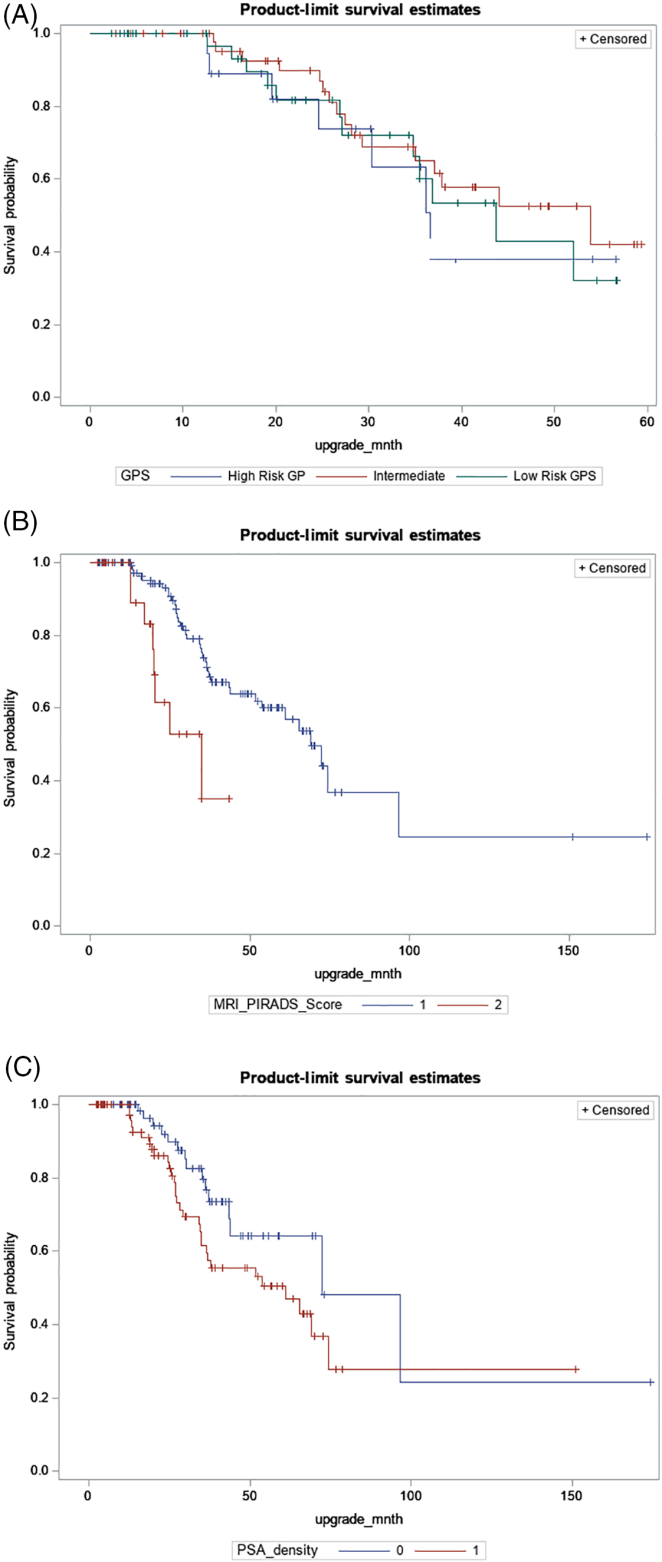
Kaplan‐ Meier Curves for biopsy upgrade free survival by (A) Different Genomic Prostate Risk Groups, (B) MRI PI‐RADs Score, with ≤3 grouped as 1 versus MRI PI‐RADs Score > 3 grouped as 2, and (C) PSA density ≤ 0.09 grouped as 0 versus PSA density > 3 0.09 grouped as 1. Categories were based on the median PSA Density. Log‐rank test is a nonparametric test comparing the upgrading times between two groups. Censored subjects are indicated as tick marks

Univariable analysis was done by Chi‐squared and fisher's exact test for categorical data and Mann Whitney test for continuous data Time‐to‐event outcomes were estimated using the Kaplan–Meier method and log‐rank test according to GPS risk category, mpMRI PI‐RADS scores and PSAD (see Figure 2). Multivariable cox proportional hazard regression was used to investigate the effect of PSAD, PI‐RADS score and GPS on upgrading. The concordance probability in the presence of censored data was calculated and comparison of the discriminative power of risk prediction models using Harrell's concordance index (HCI) was also analyzed. Harrell's concordance index is used as a broad index for the validation of predictive ability in our survival model. It is a representation of the area under the curve from Receiver Operator Curve (ROC) analysis, and a frequently used evaluation metric measuring the goodness‐of‐fit in survival models. Generally, a value over 0.7 indicates a good model.

A *p*‐value of .05 was considered statistically significant. SAS 9.4 software (SAS Institute Inc., Cary, North Carolina, USA) and STATA v14 were used for all statistical analyses.

## 3 RESULTS

The overall rate of upgrading was 31.9% (n = 46). Median time to upgrading between diagnostic and upgrade biopsy was 29.6 months (IQR 16.7, 48.7). Baseline characteristics are summarized in Table 1. The only statistically significant difference between non upgraded and upgraded groups was PSAD (0.08 ng/ml^2^ vs. 0.12 ng/ml^2^ in no vs. upgrading, *p* = .005). Mean GPS was 22.05 in the overall cohort. (20 no upgrading vs. 22 upgrading, *p* = .2044). Although there were more high genomic risk patients in the upgraded group (20.5% vs. 17.86%, *p* = .7306), this difference did not reach statistical significance in this cohort. The upgrading group had a higher rate of high‐risk MRI lesions, but again this did not reach statistical significance (no upgrading 23.47% vs. upgrading 26.09%, *p* = .7328). Radiogenomic analysis did not demonstrate any significant association between PI‐RADS score and GPS (*p* = .0632). Mean GPS was 22.3 (14.0, 26.0) for low risk PI‐RADS lesions, whereas mean GPS was 21.04 (10.0, 27.0) for high risk PI‐RADS lesions. Table 2 demonstrated PI‐RADS GPS risk group association (*p* = .0632).

**TABLE 1:** Baseline characteristics of the active surveillance cohort

Covariates	All (144)	No upgrading (98)	Upgrading (46)	*p*‐value
Age (years)	62.1 (58.4, 68.3)	61.32 (58.14, 67.37)	62.45 (59.08, 68.46)	.1884
Race, n (%)				.6079
African American	9 (6.25%)	6 (6.12%)	3 (6.52%)	
Caucasian	125 (86.81%)	83 (84.69%)	42 (91.3%)	
Asian	3 (2.08%)	3 (3.06%)	0	
Unknown	7 (4.86%)	6 (6.12%)	1 (2.17%)	
PSA (ng/ml)	5.0 (3.45, 6.18)	4.65 (3.10, 5.80)	5.36 (4.37, 7.69)	.0063
Prostate volume, (ml)	46.0 (33.0, 62.0)	47.0 (33.4, 61.0)	44.0 (32.2, 63.0)	.4094
PSA density	0.09 (0.06, 0.15)	0.08 (0.06, 0.13)	0.12 (0.07, 0.21)	.005
PI‐RADS Score				
1–2–3	118 (81.94%)	80 (81.63%)	38 (82.61%)	.8871
4–5	26 (18.06%)	18 (18.37%)	8 (17.39%)	
Gleason score				.2304
3 + 3	141 (97.92%)	95 (96.94%)	46 (100.0%)	
3 + 4	3 (2.08%)	3 (3.06%)	0 (0.0%)	
Number of positive cores	2.0 (1.0, 4.0)	2.0 (1.0, 3.0)	2.0 (1.0, 4.0)	.1610
Max % of core involvement	15.0 (5.0, 30.0)	15.0 (5.0, 30.0)	17.5 (9.0, 30.0)	.8662
Genomic prostate score	21.0 (14.0, 27.0)	20.0 (12.0, 27.0)	22 (15.0, 28.0)	.2044
GPS risk				
Low	44 (35.77%)	32 (38.10%)	12 (30.77%)	.7306
Intermediate	56 (45.53%)	37 (44.05%)	19 (48.72%)	
High	23 (18.7%)	15 (17.86%)	8 (20.51%)	

**TABLE 2:** PI‐RADS GPS association

	GPS Risk Group		
MRI PI‐RADS	Low	Intermediate	High
1–2–3 (Low)	30	49	18
4–5 (High)	14	7	5

In univariate cox proportional hazard regression analysis, the factors associated with increased risk of biopsy upgrading were PSA (HR = 1.30, CI 1.16–1.47, *p* = <.0001), PSAD (HR = 1.08, CI 1.05–1.12, *p* = <.0001) and high PI‐RADS score (HR = 3.51, CI 1.56–7.91, *p* = .0024). In multivariate cox proportional hazard regression analysis, PSAD (HR = 1.09, CI 1.05–1.13, *p* = <.0001) and high PI‐RADS score (HR = 4.11, CI 1.79–9.44, *p* = .0009) were the only factors associated with upgrading. (Table 3). Kaplan–Meier curves and log‐rank test (*p* = .7223) stratified by the GPS score, PI‐RADS and PSAD are shown in Figure 2.

**TABLE 3:** Univariate and multivariate cox regression analysis to predict upgrading during active surveillance. Two models were built using MRI variables (Model 1) and MRI variables in addition to GPS score (Model 2)

	Univariate cox			Multivariate cox regression using MRI variables (Concordance Index = 0.692)		Multivariate cox regression using MRI variables in addition to GPS score (Concordance Index = 0.71)	
Covariates	HR (95% CI)	C index	*p*‐value	HR (95% CI)	*p*‐value	HR (95% CI)	*p*‐value
Age, y	1.04 (1.00, 1.08)	0.611	.056				
PSA ng/ml	1.30 (1.16, 1.47)	0.674	<.0001				
PSAD ng/ml2	1.08 (1.05, 1.12)	0.656	<.0001	1.09 (1.05,1.13)	<.0001	1.10 (1.06, 1.14)	<.0001
Lesion on MRI	3.40 (1.69, 6.87)	0.611	.0006				
PIRADs score		0.582	.0024				
1,2,3	Ref						
4,5	3.51 (1.56, 7.91)			4.11 (1.79, 9.44)	.0009	4.21 (1.75, 10.13)	.001
MRI Prostate volume, ml	1.0 (0.99, 1.01)	0.463	.9379				
GPS risk category		0.50					
1	Ref						
2	0.97 (0.47, 2.01)		.9407			1.14 (0.54, 2.38)	.7292
3	1.17 (0.48, 2.88)		.7260			0.97 (0.39, 2.42)	.9500
GPS risk score	1.01 (0.99, 1.04)	0.55	.3276				

A cox regression model combining these three clinical features (PSAD ≥0.15 ng/ml^2^ at follow up biopsy, PI‐RADS Score and GPS) yielded a Harrell's concordance index of 0.71 for the prediction of pathological progression, whereas the concordance index for PI‐RADS score alone was 0.582, GPS alone 0.50 and for PSAD alone was 0.65. A model with both PSAD and PI‐RADS had a concordance index of 0.692, demonstrating the addition of GPS did improve predictive accuracy.

## 4 DISCUSSION

The results of this study show, with nearly 5 years follow up, PSAD has more value than both mpMRI alone and GPS alone as a tool predicting upgrading on biopsy. With specific reference to PSAD, the results correlate well with previous studies in the literature, which have also reported a PSAD greater than 0.15 is associated with upgrading.^12^, ^13^ The results of this study support the utility of mpMRI in AS, not only because high PI‐RADS score was a significant predictor of upgrading, but also by measurement of MRI prostate volume to calculate PSAD which is more accurate than TRUS volume measurements,^14^ and allowing improved diagnostic accuracy on follow up biopsies. Walton Diaz et al. performed a comparative analysis of 12 core standard biopsy and targeted biopsy in the AS setting, demonstrating that targeted biopsy increased the detection of upgrading from 13.8% to 29.3%.^15^ These results do not mean that GPS should not be used in AS, since the unified model combining PSAD, PI‐RADS score and GPS yielded the highest predictive ability compared to each test individually.

Our results are discordant with the results of Cedars et al. regarding the predictive ability of GPS. In their series of 111 patients, a gradual increase in GPS between biopsies was seen, and the increase in GPS from initial biopsy was associated with an increased rate of upgrading at follow up biopsy.^16^ With comparable follow up to Cedars et al.’s patients, in this cohort, GPS was higher in the upgraded cohort, but the association did not reach statistical significance. Kornberg et al. also found GPS correlates with biopsy upgrade in 131 patients under AS,^17^ but interestingly, Lin et al. demonstrated that GPS score for patients in AS was not associated with Adverse Pathology (AP) for those who converted to treatment, which the majority of upgraded AS patients would be offered.^7^, ^17^ For Cedar et al., the rate of grade reclassification was 44%, which is significantly higher than this cohort (31.9%), but on a par with the results of van Hemelrijck, in a multinational multicenter study on 10 296 patients, who reported 43.6% upgrading at 5 years follow up.^3^, ^7^ The reasons for the higher upgrade rates in these studies are discussed in more detail later in this section but are likely to be multifactorial, including differing inclusion/exclusion criteria, differing use of markers of stable disease as well as varying AS program protocols across different countries and centers.

With regards to mpMRI lesion characteristics, a recent analysis by Gallagher et al. demonstrated results supporting those of our study, although their protocol for MRI reporting was different. Gallagher et al. did not use the PI‐RADS system but rather MRIs were classified as having “high risk” or “moderate risk” lesions or “no lesion”. They reported worse progression free survival in patients with high risk lesions compared to patients with no lesions (HR: 3.5, *p* < .001). Furthermore, they demonstrated that patients with a visible lesion at confirmatory biopsy had a higher risk of upstaging compared to no lesions (OR: 4.1, *p* < .001). The same analysis was not carried out for comparison in this cohort, as confirmatory biopsy was not done on patients without lesions on mpMRI. Finally, they showed in a sub‐cohort of patients without any mpMRI lesions, performing a biopsy did not change the rate of progression (12.5% with confirmatory biopsy vs. 13.3% without confirmatory biopsy, *p* = .87),^18^ which supports our rationale of foregoing biopsy in patients with pristine mpMRIs. Another recent study confirmed that baseline mpMRI PI‐RADS score correlates with biopsy upgrade. After 2 years follow up in AS, Fujiwara et al. showed 37% of patients with PI‐RADS 3–5 lesions on baseline mpMRI upgraded compared to only 12% of patients with PI‐RADS 1–2 lesions.^19^ Prior to this, classification of mpMRI progression on serial MRIs for AS patients led to the Prostate Cancer Radiological Estimation of Change in Sequential Evaluation (PRECISE) score of 1–5, with 1 being regression, 3 being stable, and 5 being progression to ECE or SVI.^20^ In a study of 533 patients with 76 months follow up, the PRECISE score was shown to be an effective predictor of disease progression, with only 5% of patients scoring 1–3 on serial MRIs having biopsy upgrade, compared to 61% of patients with scores 4–5 upgrading on subsequent biopsy.^21^ Further supporting the use of MRI, the Active Surveillance Magnetic Resonance Imaging *Study* (ASIST) trial, which was a randomized study comparing systematic biopsy versus MRI and targeted and systematic biopsy in 259 AS patients, showed baseline MRI prior to confirmatory biopsy, reduced subsequent conversion from AS to treatment by 50% after only 2 years follow up.^22^ As with prediagnostic use of MRI, the ultimate goal would be MRI having sufficient accuracy in diagnosis to replace serial biopsy in AS follow up. However, many of these studies are performed in tertiary referral centers, and the reality for most practicing Urologists is that day to day MRIs in AS programs may be excellent but not yet of sufficient quality to replace histopathological examination. Until such time when that is the case, other parameters highlighted in this study will remain valuable tools.^23^

Exploring radiogenomic associations may also support the growing utility of mpMRI in the AS setting. In this cohort there was no association between GPS and either low or high risk PI‐RADS lesions on baseline MRI (see Table 2.). Conversely, in a similar analysis, Leapman et al. reported GPS scores in different PI‐RADS group in an AS setting, demonstrating a GPS PI‐RADS association only in patients with csPCa (Gleason 3 + 4 or higher).^24^ Other studies have also demonstrated a correlation between MRI PI‐RADS lesions representing csPCa and GPS, including Kornberg et al. discussed above.^17^, ^25^ Examining prostate tissue from RP specimens, the authors of this study have previously demonstrated differential genomic pathway expression between PI‐RADS 3 and PI‐RADS 5 lesions, although that study used a different genomic assay.^26^ Interestingly a recent systemic review and bioinformatic analysis of 32 articles concluded that MRI visible prostate cancer is characterized by genomic features that correlate with aggressive disease including PTEN loss, DNA damage repair, proliferative signaling and higher genomic classifier scores (including GPS).^25^ This supports the role of MRI in predicting disease states in AS patients, but clearly more study is required.

The GPS score in this study was not significantly associated with biopsy upgrade or with high risk PI‐RADS 4 or 5 lesions. It is possible there is some time dependence for the accuracy of the GPS score, so as tumor biology deteriorates over time in AS, GPS score at diagnostic biopsy becomes less representative of tumor risk. The median time between diagnostic and upgrade biopsy was 29.6 months (IQR 16.7, 48.7), whereas, for example, in Cedars et al., the mean time between first and second biopsy (which would have been the upgrade biopsy) was only 14 months (IQR 12–23). Also, in this cohort, there were only three patients with Gleason 7, and only 26 patients with PI‐RADS 4 or 5 lesions, which suggests this AS program is conservative in recruitment with a low threshold for offering radical treatment at the time of diagnosis. Given that in the studies discussed above,^17^, ^24^, ^25^ GPS correlated with MRI only for those with csPCa and there was a predominance of lower risk patients in this cohort, not only does this potentially explain the low conversion rate in comparison to other studies, but also supports the notion this study may be underpowered to show any significant association of GPS with upgrading.

Despite the limitations of comparing studies with different mpMRI and AS protocols, there is a reasonable consensus that mpMRI does has value in AS, albeit when combined with other parameters such as PSAD and GPS score.^23^ However, analysis of the Surveillance, Epidemiology, and End Results (SEER) database showed that only 13% of patients received mpMRI among AS patients. Furthermore, use of mpMRI in AS management is associated with an annual $447 increase in Medicare spending per year, and therefore, mpMRI should be optimized to maximize the value of the test.^27^ There is also concern regarding the increased healthcare cost associated with the use of GPS, although by altering decision‐making towards AS in lower risk patients, and it may become cost effective by decreasing costs associated with overtreatment.^28^

This study is a single center study with retrospective design and so inherent selection bias. Also, the AS protocol for this study differs with most studies in that confirmatory biopsy was not done in every patient, and, in the absence of any changes in disease parameters, repeat biopsy is not done until 3 years after diagnosis. All patients diagnosed at this institution were diagnosed with mpMRI fusion biopsy unless their mpMRI was without lesions, and patients received confirmatory biopsy only if they were diagnosed at an outside institution without targeted biopsy and their follow up mpMRI showed lesions suggestive of csPCa. This practice aims to reduce the number of biopsies. Finally, all mpMRI studies were reported prospectively as part of routine clinical workflow at different institutions and only a portion of studies was re‐reported (n = 75) by one reader unaware of PSA, PSAD, GPS and biopsy findings, which means differences in MRI acquisition protocol and MRI scanners were present during the study period. On the other hand, this study has a reasonable size and is a single surgeon series, so treatment decision‐making did not vary within the cohort.

## 5 CONCLUSION

PI‐RADS score of the baseline mpMRI and GPS were shown to behave some potential for predicting upgrading at nearly 5 years follow up in this single center tertiary referral center AS cohort, however their predictive ability was inferior to PSAD. mpMRI and GPS remain useful tools in AS, however, their optimal use is yet to be determined. Moreover, with the development of scoring systems such as “PRECISE”, the utility of MRI in AS may be expanding. Nevertheless, the combined use of PSAD, GPS and mpMRI yields the highest predictive ability for identifying pathologic upgrading at follow up biopsy in patients undergoing AS in this study, and on that basis, the use of all three parameters when recruiting patients with newly diagnosed PCa to AS programs is recommended.

## ETHICAL STATEMENT

This analysis was institutional review board (IRB) approved with appropriate consent obtained for all patients included.

## CONFLICT OF INTEREST

Z.D. is Medical Director and stock owner (by shares) of MedTech Holdings Ltd. AKT as of 2020 listed as company type, relationship type and financial. Urethral Catheterless Radical Prostatectomy, Patent, No. DNA Based Bicistronic Vectors with Inducible and Constitutive Promoters ‐ ID#: 16060, Patent, No. High Intensity Focus Ultrasound and CPG‐Brachyurys iRNA for Treatment of Prostate Cancer ‐ ID# 160403, Patent, No. Patent for a Catheterless Device and Approach, Patent, No. *Promaxo, Leadership position, Yes. *Promaxo, Equity Ownership, yes. Global Prostate Cancer Research Foundation, Leadership position, No. Kalyani Prostate Cancer Institute, Leadership Position, No. Prostate Cancer Foundation, Leadership Position, No. Roivant, Consultant, No. Blank Family Foundation, Grant, Yes. Intuitive Surgical, Scientific Study or Trial, Yes. Department of Defense (DOD), Scientific Study or Trial, Yes. AxoGen, Inc., Scientific Study or Trial, Yes. Oncovir, Inc ‐ Poly ICLC, Scientific Study or Trial, Yes. National Institute of Health (NIH/DHHS), Scientific Study or Trial, Yes. National Cancer Institute, Scientific Study or Trial, Yes. National Institute on Drug Abuse, Scientific Study or Trial, Yes. Dr. Ash Tewari (the Principal Investigator in this study and Chairman of Milton and Carroll Petrie Department of Urology at the Icahn School of Medicine at Mount Sinai) owns equity in the form of stock certificates in Promaxo, for which he serves as an advisor. Promaxo is a privately traded company which develops MRI technology with a focus on prostate cancer. Kite Pharma, Scientific Study or Trial, Yes. Lumicell, Inc, Scientific Study or Trial, Yes. Dendreon, Scientific Study or Trial, Yes. *PROMAXO: Common Stock Certificate VALUE: 51,205% SHARE RELATED PARTY: 0.63; Intuitive – no salary/see referenced COI’s; Promaxo – no salary/investment – see referenced COI’s; Kite Pharma: Serve as PI. Industry funded for research procedures. Site PI performance – no salary; Poly ICLC: serve as site PI. Phase I Study of IN SITU Autologous Vaccination Against Prostate Cancer With Intratumoral and Systemic HILTONOL (POLY‐ICLC) Prior to Radical Prostatectomy. Product provided free of charge – no other funding ‐ no salary.

## AUTHOR CONTRIBUTIONS

**T.B.:** Conceptualization; methodology; writing‐original draft; writing‐review & editing. **P.R.:** Data curation; formal analysis; methodology; writing‐original draft; writing‐review & editing. **Z.D.:** WritingOriginalDraft; WritingReviewEditing. **R.R.:** Data curation; formal analysis; investigation; methodology. **S.P.:** Data curation; formal analysis. **S.S.:** Formal analysis; investigation; methodology; writing‐review & editing. **D.K.:** Data curation; formal analysis; investigation; methodology. **R.W.:** Formal analysis; investigation; methodology. **H.B.:** Formal analysis; investigation; methodology. **I.J.:** Formal analysis; investigation; methodology; writing‐review & editing.

## Data Availability

The data that support the findings of this study are available from Prof. A. Tewari but restrictions apply to the availability of these data, and so are not publicly available. Data are however available from the authors upon reasonable request and with permission of Prof. A. Tewari.
